# The Bayne Antivivisection Bill

**Published:** 1911-05

**Authors:** 


### The Bayne Antivivisection Bill.

If we were under the dire necessity of giving support to either of the antivivisection bills now before the New York Legislature we would favor the Pollock rather than the Bayne bill, for the reason that an open enemy is preferable to a secret one. The framers of the Pollock bill are frankly opposed to animal experimentation and the bill is designed to put every restriction possible, short of absolute prohibition, upon it. The framers of the Bayne bill are ostensibly open-minded inquirers seeking the truth. But the real sentiments of those behind the measure are well known and no one with any knowledge of the facts can doubt as to what the finding of the necessarily partisan committee of investigators would be. The expenses of the inquiry are to be met, not by the State but by private individuals or societies. The money will not be contributed by those already convinced of the value of animal experimentation, but will of course be supplied by the antivivisectionists. The commission will, therefore, be in the employ of fanatics, and will naturally start with a bias in favor of those whose money they are spending. But even if they were so superhuman as to see things as they really are and to report that there was no cruelty in the practice of animal experimentation, the principle would remain bad. As well might the Christion Scientists demand the passage of a bill for an investigation of hospital surgeons in order to determine whether their operations are justifiable. There are malpractice laws and there are laws preventing cruelty to animals. If surgeons cut up their patients unnecessarily they are answerable to the malpractice laws; if experimenters inflict cruelty upon animals they can be punished by the existent laws. The Bayne bill should be defeated. It is pernicious.—*New York Medical Record.*

